# Response of peanut (*Arachis hypogaea* L.) plant to bio-fertilizer and plant residues in sandy soil

**DOI:** 10.1007/s10653-022-01302-z

**Published:** 2022-06-13

**Authors:** T. M. S. El-sherbeny, Abeer M. Mousa, Mostafa A. Zhran

**Affiliations:** 1 Nuclear Research Center, Plant Research Department, Egyptian Atomic Energy Authority, Cairo, Egypt; 2 Nuclear Research Center, Soil and Water Research Department, Egyptian Atomic Energy Authority, Cairo, 13759 Egypt

**Keywords:** *Azotobacter *sp., *Bradyrhizobium *sp., ^15^N labeled, N_2_-fixation, Plant residues, Peanut

## Abstract

Nitrogen (N) fertilizer has been intensively used to improve peanut productivity. However, the high cost of N fertilizer, and the need for sustainable alternative fertilizer sources have increased the strategic importance of nitrogen fixation (NF). Thus, field experiments were conducted in an experimental farm with a drip irrigation system, at the Atomic Energy Authority, Inshas, Egypt, in order to measure the impact of efficiency symbiotic *Bradyrhizobium *sp. and asymbiotic *Azotobacter *sp. on NF, from air and soil, in the presence or absence of plant residues on the growth and yield of peanut plant. All treatments received nitrogen fertilizer at a rate of 72 kg N per hectare. Nitrogen dose was applied using ammonium sulphate ^15^N labeled of 10% atom excess from the peanut. Results indicated that the application of *Bradyrhizobium* sp. with plant residues significantly increased fresh and dry weight/m^2^, pod and seed weight/plant^−1^,100- seed weight, and biological yield kg ha^−1^, where the highest mean values of seed yield (4648 and 4529 kg ha^−1^), oil % (52.29 and 52.21%), seed protein percentage (16.09 and 15.89%), as well as nitrogen derived from air (63.14 and 66.20%) in the first and second seasons were recorded under the application of *Bradyrhizobium* sp, respectively. *Bradyrhizobium *sp. inoculation showed nearly close portions of Ndfa to those recorded with *Azotobacter *sp., in both the presence and absence of plant residue application through the two seasons. The investigated yield signs and their properties were significantly enhanced by bacterial inoculation with plant residue application. The present study shows that both possibility of NF of peanut, and nitrogen uptake in the soil are enhanced by field inoculation with effective *Bradyrhizobium *sp. with plant residue application. In practice, inoculation is a great strategy to improve soil fertility for subsequent planting, since it helps boost the import of nitrogen from plant biomass into the soil.

## Introduction

Peanut (Arachis *hypogaea* L.) is an essential and economical oil, food, and feed crop for the world. It originated in the Central American region, spread to other areas in the world, and developed in nearly all tropical and sub-tropical countries. Its most significant producers are India, China, the USA, and West Africa (Bhatti et al., 2010; Krishnappa et al., 1999). Peanuts are often enriched with health benefiting nutrients that can be beneficial for human health. Its seed contains about 25–30% absorbable protein, 45–50% oil, 20% carbohydrate, and 5% fiber and slag, making it a crucial source of nutrition for humans (Ahmad & Rahim, 2007). Additionally, peanut cake is utilized as animal feed and organic manure (Shah et al., 2012). Peanut plant is effectively developed in recently recovered sandy soil in Egypt, which experiences insufficiency or inaccessibility to most of the micronutrients, because of its suitability for growth in sandy soil. Egypt’s recently reclaimed sandy soils have been recommended for their poor soil inclusion, low natural emissions, reduced water retention, and reduction of nutrient production. However, the response of peanut (*Arachis hypogaea* L.) plant grown in sandy soil to biofertilizer management remains poorly understood.

Organic fertilizers diminish pollution, and expand soil development by influencing the physical and natural properties of the particular soil (Hosam El-Din, 2007, Zeidan et al., 2009 and Zaki et al., 2012). Biofertilizer, like *Azotobacter,* changes the ordinarily airborne nitrogen into ammonia. Ammonia penetrates into the particular root zone, as well as the half nitrogen needs connected with accessible for root use, and changes the blocked off calcium phosphate to available form (Ahmed et al., 2010). For ideal plant growth, nutrients should be changed and ought to be adequate for plant (Ayoola, 2010). Nonetheless, a large number of these resources come in the unavailable form, out of which a little part is released every year through biological activity, and chemical processes. Furthermore, 60–90% of the whole applied fertilizer is lost, while the remainder of the 10–40% is utilized by plants. Hence, biofertilizers could be an important component of nutrient management systems, for sustaining agricultural productivity, and a healthier environment (Adesemoye & Kloepper, 2009). Biofertilizers can impact crop productivity, since they may increase plant growth and quality of crops, and reduce the expense of fertilizer and pesticide application (Chen, 2006). They keep the soil environment full of all sorts of required nutrients for the plant, such as N, phosphate, and potassium mineralization or solubilization. When applied with seed or soil inoculants, they improve nutrients cycling and contribute to crop productivity.

The biological nitrogen fixation (BNF) is probably the main natural biological process after photosynthesis (Unkovich, 2013), which is highly relevant to sustainable agriculture (Udvardi & Poole, 2013). Consequently, it represents the critical form of nitrogen input for many terrestrial ecosystems. BNF plays an essential role in soil improvement. Leguminous plants and rhizobia together form an asymbiotic relationship (Freiberg et al., 1997; Zahran, 2001). The symbiotic relationship among *Rhizobium* and legumes is the principal supply of fixed nitrogen. Symbiotic bacteria infect the legume roots and form nodules (West et al., 2002). Zarei et al. (2012) revealed that soybean inoculated with *Bradyrhizobium japonicum*, *Bacillus megaterium,* and 50% triple superphosphate has proper balance between vegetative and reproductive growth, and complete developmental stages of seeds. This status can be made when the fundamental components for vegetative growth (nitrogen) are adjusted with the essential elements for reproductive growth (phosphorus). *Bradyrhizobium japonicum*, and *Bacillus megaterium* increase seed yield by providing macro and micro nutrients for plant growth.

Symbiotic nitrogen fixation is among the essential biological processes for development of sustainable agriculture, by which the atmospheric nitrogen transforms into ammonia with the assistance of a key enzyme called nitrogenase (Oldroyd et al., 2011; Udvardi & Poole, 2013). It is accomplished by bacteria in the cells forming organs, and the nodules on roots of numerous leguminous plants. The peanut growth process requires the absorption of different elements, especially Fe and Mo. Like legumes, peanut can fix dinitrogen (N_2_) by establishing mutualistic symbiosis with compatible rhizobia strains, basically from the genus Bradyrhizobium (Zhang et al., 2015). Molybdenum-iron protein could be the active site of nitrogenase (Keable et al., 2018). Previous studies have shown that Fe deficiency causes serious chlorosis, inhibiting the growth of peanut seedlings, and decreasing the concentration of soluble Fe and chlorophyll in peanuts (Kong et al., 2015). Asymbiotic nitrogen-fixing bacteria (free living, associative, and endophytes) are Cyanobacteria, *Azospirillum*, *Azotobacter*, *Gluconacetobacter diazotrophicus,* and *Azocarus*. (Bhattacharyya & Jha, 2012). As a result of the inefficiency of suitable carbon and energy sources for free-living, their role in nitrogen fixation is recognized as minor (Wagner, 2011). *Azotobacter* further develops plant growth, by synthesizing IAA and other growth-promoting substances that promote plant growth, cell division, and breaking the special dominances, hence, encouraging the photosynthesis, and assimilating accumulation (Ahmad et al., 2005). Thus, the aim of this study is to evaluate the effect of asymbiotic bacteria *Azotobacter *sp. and symbiotic bacteria *(Bradyrhizobium *sp.) on enhancing the nitrogen fixation on peanut yield. Applied plant residues are incorporated to the soil three month prior to peanut cultivation. As well as reduce environmental hazards of using chemical fertilizers to improve peanut yield.

## Materials and methods

### Microbial inoculations

*Azotobacter *sp.* and Bradyrhizobium *sp. were obtained from the Agricultural Research Center (ARC), Ministry of Agriculture Giza, Egypt. For the symbiotic strain, the Bradyrhizobium sp. strain was cultured in yeast extract mannitol broth (Vincent, 1970), until it reached the late logarithmic phase of growth. The inoculum was applied at an initial population level of 10^8^ cfu/ml. For the asymbiotic strain, *Azotobacter *sp. was maintained in modified Ashby’s nitrogen-free liquid medium (Abd El-Malek & Ishac, 1968) separately, for 3–5 days at 28 °C with agitation (100 rpm). The inoculum was applied at an initial population level of 10^8^ cfu/ml.

### Experiment in vitro study

The aim of the experiment is to study the effect of molybdenum (Mo) and iron (Fe) on the nitrogen fixing activity of *Azotobacter *sp*. and Bradyrhizobium *sp. Various concentrations of the individual of sodium molybdenum (Na_2_MoO_4_), and of iron sulphate (FeSO_4_) were used. The highest rate used for the solution was (10 mg/l), while the medium was modified Ashby’s N-free medium. The pH of the medium was adjusted to 7 ± 0.2 before autoclaving at 121 °C for 15 min. One ml of 10^8^ bacterial suspension was inoculated to 10 ml of the modified Ashby medium in a test tube with a cotton plug, for 7 days, till the mid-exponential phase at 28 °C. Then, the cotton plug was replaced with a rubber stopper. 1 ml of the atmosphere (10%) was replaced with acetylene by injection, then incubation continued for 4 h. The 1 ml gas sample was removed using a 1 ml syringe, and the ethylene concentration was measured by gas chromatography. The ability of *Azotobacter *sp*. and Bradyrhizobium *sp*.* to fix atmospheric nitrogen was measured based on the ability of *Azotobacter sp. and Bradyrhizobium sp.* nitrogenase complex enzyme to reduce acetylene (C_2_H_2_) to ethylene (C_2_H_4_) (Volkohon, 2010). This experiment was conducted to point out the best concentration of Mo and Fe for nitrogenase activity to be selected before application in the field experiment.

### Field experiment

Two field experiments were carried out in the experimental farm at the Nuclear Research Center, Atomic Energy Authority, Inshas, Egypt (latitude, 30° 24′ N; longitude, 31° 35′ E; elevation, 20 m), during the two growing seasons of 2017 and 2018, in order to study the impact and efficiency of three bio-fertilizer treatments; i.e., symbiotic *Bradyrhizobium *sp., asymbiotic bacteria *Azotobacter *sp., and uninoculated (control), and two plant residue treatments; i.e., applied plant residues, and without plant residues on growth, yield, and its components on peanut plant and nitrogen fixation from air and soil. Soil samples were collected from plough layer (0–30 cm) of the field, air dried, and sieved to pass through a 2 mm sieve. The physical and chemical properties of the experimental soil sample are presented in Table 1.

**Table 1:** Physiochemical properties of the experimental soil in both seasons

	Texture grade	pH	Mechanical analysis								
			Sand (%)	Silt (%)	Clay (%)	Organic matter (%)	Organic carbon (%)	N (mg/kg)	P (mg/kg)	K (mg/kg)	CaCO_3_ (mg/kg)
1st season	Sandy soil	7.46	91.7	5.9	2.4	0.33	0.19	18	5.9	169	0.17
2nd season		7.39	91.1	6.2	2.7	0.35	0.20	21	6.1	149	0.17

Incubation period in soil (days in soil)	N (%)	C %	Organic carbon %	C:N ratio
0 days	0.5	78	45	90%
60 days (before planting)	1.36	31	17.9	13.1%

Plant residues (casuarina leaves, ficus leaves, rice straw, and wheat straw) were grained with a suitable mill, then incorporated into the soil during preparation for sowing. They were added at a rate of 36-ton ha^−1^, and calculated based on N% in plant residues. Three cellulolytic bacterial strains (*Achromobacter spanius, Bacillus amyloliquficinous, and Stenotrophomonas maltofilia*) were obtained from the researchers’ laboratory of the Microbiology lab, at the Soil and Water Research Department, Nuclear Research Center, EAEA. After, the three cellulolytic bacterial strains were incubated with plant residues by adding 200 ml × 10^8^ cfu ml^−1^ from cellulolytic bacterial strains for each plot. The bacterial strains and plant residues were incorporated into the soil three month prior to peanut cultivation, and soil was kept wet until planting.

Peanut (*Arachis hypogaea*) “GIZA 6” seeds were provided by the Agricultural Research Center, Egypt. The peanut seeds were planted on May 15th, 2017 and May 13th, 2018 respectively, on ridges 60 cm apart, in hills 20 cm apart. At thinning (15 days from sowing), one peanut plant was left in hill. Moreover, sesame (*Sesamum indecum L*) plant C.V shandaweel-3 seeding was mod, at the same time, as reference crop in sand soil. The amount of nitrogen derived from the atmosphere (Ndfa) was estimated using non-legume crops as reference crop. The drip irrigation system was prepared especially for this purpose using neutron moisture to water irrigation depth, in order to limit rooting zone during the growth period of the upper 30 cm in depth. Each plot area was 12 m^2^ (4 m long in 3 m wide). One milliliter of *Azotobacter *sp., and *Bradyrhizobium *sp*.* was added per seed. This step was repeated after 3 days to ensure a sufficient bacterial population. These two inoculants *Azotobacter sp. & Bradyrhizobium *sp*.* were separately applied to seeds.

Nitrogen fertilizer was applied at the rate of 72 kg N ha^−1^ in the form of ammonium sulfate, as activated dose after 15 days from planting as a starter dose. Nitrogen fertilizer (^15^N-ammonium sulfate enriched with 10% atom excess) was applied at the same rate in a microplot (1 m × 0.6 m) to be 0.60 m^2^. Phosphate fertilizer (480 kg ha^−1^) was added in the form of calcium phosphate (15.5% P_2_O_5_), and 240 kg ha^−1^ in the form of potassium sulphate (48% K_2_SO_4_), as recommended by the Ministry of Agriculture in Egypt. Molybdenum (Mo) and iron (Fe) were added to the soil one time after 10 days from transplanting during the two seasons, by which Ferric chloride (FeCl_3_–6H_2_O) was at dose 50 g ha^−1^, and Ammonium molybdate tetrahydrate (NH_4_)6Mo_7_O_24_·4H_2_O at dose 1 g ha^−1^.

### Measurements

After 75 days from sowing (DFS), fresh weight (g/m^2^) and dry weight (g/m^2^) were recorded. Plant samples were immediately sent to laboratory, and standardized to 10 cm-depth removing the excess material (dirt and roots). Roots were washed, nodules were separated, and the total number of nodules per plant was determined. At harvest, plants were randomly taken from the plot of each experimental unit to determine yield components, namely pod weight (g plant^−1^), (seed weight g plant^−1^), 100-seed weight (g), and shelling percentages. Straw yield (kg ha^−1^), seed yield (kg ha^−1^), biological yield (kg ha^−1^), and oil percentage (Harwood, 1984), as well as total carbohydrate percentage (Dubois et al., 1956), and seed and straw protein percentage (Mozingo et al., 1988) were recorded. ^15^N stable isotope technique provided the opportunity to quantify the fraction of N derived from different sources with the exact values, which facilitated the recognition of the best strategy of N management under low input agriculture. The emission Spectrometer (Jasco Model-150) was used for the ^15^ N-estimation. Calculation of the ^15^N atom excess in plant materials was obtained using the ^15^N atom % provided during the analysis, and atmospheric natural ^15^N abundance (0.3662) by applying the equation. The calculations in this paper were in accordance with the International Atomic Energy Agency.

$$^{{{15}}} {\text{N\,atom }}\% {\text{ excess}}\, = \,^{{{15}}} {\text{N\,atom }}\% {-\!\!-}{\text{natural}}^{{{15}}}\, {\text{N\,abundance}}$$

$${\text{N}} - {\text{uptake}} = {\text{N }}\% \times {\text{dry}}\;{\text{weight}}$$

$$\% {\text{Ndf}}f = \frac{{\% 15_{{\text{N}}} {\text{Atom excess of sample}} }}{{ \% 15_{{\text{N}}} {\text{Atom excess of fertilizer}}}} \times 100$$

$$\% {\text{Ndfs}} = \% {\text{Ndff}} - 100$$

$$\% {\text{Ndfa}} = 1 - \frac{{{\text{atom \% 15}}_{{\text{N}}} {\text{ excess N}}_{{2}} {\text{ - fixing plant }}}}{{{\text{atom \% 15}}_{{\text{N}}} {\text{ excess reference plant}}}} \times 100$$

$$N_{2} {\text{fixed}} = \frac{{\% {\text{Ndfa}}\,\times\, {\text{total }}N {\text{in fixing crop}} }}{ 100}$$

### Statistical analysis

The experiments were designed in a randomized complete block design in three replicates. The in each season were statistically analyzed, and the least significant differences were in accordance with Sendecer and Cochron (1989).

## Results and discussion

### Vitro study

**Fig. 1:**
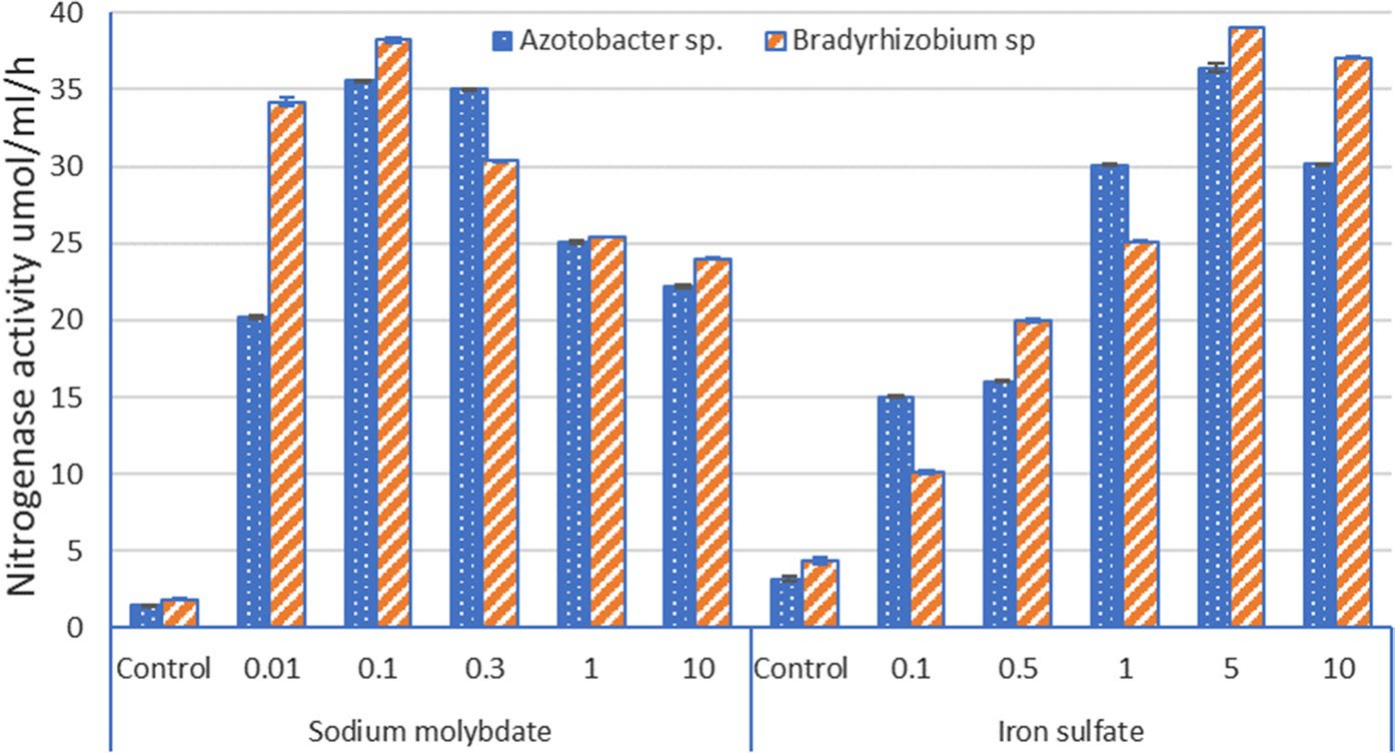
Detection of the nitrogenase activity (umol C_2_H_4_/ml/h) of *Bradyrhizobium *sp. and *Azotobacter *sp. under influence of iron and molybdate

The effect of molybdenum and iron on the activity of nitrogenase enzyme by *Azotobacter *sp., and *Bradyrhizobium* sp*.* in the medium. The maximum production of nitrogenase enzyme activity was observed with *Azotobacter* sp, and *Bradyrhizobium* sp*.* as affected by sodium molybdate and iron sulfate. Figure 1 shows nitrogenase enzyme activity with concentrations that range from 0 to 10 mg L^−1^ for both molybdenum and iron. The highest nitrogenase enzyme activity obtained with *Azotobacter *sp*.* and *Bradyrhizobium* sp was observed at 0.1 mg L^−1^. At lower concentrations, the detected amount of nitrogenase activity was also lower, yet exceeded the indices in variants with sodium molybdate. The best nitrogenase enzyme activity of *Azotobacter* sp, and *Bradyrhizobium* sp*.* was observed at concentration of iron in the medium at 5 mg L^−1^. Generally, nitrogenase activity was higher for *Bradyrhizobium* sp*.* compared to *Azotobacter* sp. Iron (Fe) and molybdenum (Mo) are necessary trace elements for plants, especially for peanut (Chun-Lun et al., 2019). Many authors have reported the utilization of sodium molybdate (Halder & Chakrabartty, 2015; Rousk et al., 2017), and iron sulfate (Zhang et al., 2015). Iron is a significant micronutrient for the symbiosis because several symbiotic proteins incorporate iron as the bacterial nitrogen-fixing enzyme (nitrogenase). Cytochromes are needed for phosphorylation in the plant, and electrons reduce the iron element of nitrogenase enzyme (Brear et al., 2013).

### Field experiment

Plant residues significantly increased yield and yield components based on the significant upsurge in the number of nodules. The kind of bacterial strain inoculation affected the amount of nodule of the peanut plant. Nodules might be clearly observed 45 days after inoculation. These results indicated that nodules present with treatment *Azotobacter* or uninoculated with or without plant residues originated from the native population in soil. Rhizobacteria, which include both symbiotic and asymbiotic bacteria, are one of the most important classes of soil microbiota that boost crop yields, particularly legume yields, in a variety of agronomic settings (Turan et al., 2017). Sarr et al. (2015) found that higher nitrogen fixation in legume crops was linked to large mature nodules, rather than a large number of small immature nodules. The application of an additional external source, such as millet straw, was discovered to increase nitrogen fixing in ground nuts together with nodule formation (Rebaka, 1993). Hence, plant residues as organic fertilizer, and inoculation with *Bradyrhizobium *sp*.* and *Azotobacter *sp. did not only increase peanut yield and its components, but also improved its nutritive value. These results might be attributed to the beneficial effect of nitrogen on metabolic processes and growth, which often reflected.

Results also revealed that the interactions between plant residues with *Azotobacter *sp, and *Bradyrhizobium *sp*.* inoculation were significant *p* < 0.05 for fresh and dry weight g/m^2^ after 75 days from sowing DFS; pod weight g/plant^−1^, seed weight g/plant^−1^, shelling percentage, and 100-seed weight per gram in both seasons are shown in Table 2. The application of plant residues as organic fertilizer with *Bradyrhizobium sp.* inoculation is observed to provide the most effective values for all the fresh and dry weights after 75 DFS ( 2080, 349 g/m^2^ and 2060, 335 g/m^2^) in the first and second seasons, respectively, compared to uninoculated plants. On the other hand, Azotobacter *sp* inoculation presented higher values for most of the fresh and dry weights after 75 DFS (1951, 331 g/m^2^ and 1915, 325 g/m^2^) in the 1st and 2nd seasons, respectively. The lowest values of fresh and dry weights were observed after 75 DFS with peanut plant, without plant residues in most treatments with inoculation and uninoculated. Plant residues with *Bradyrhizobium *sp*.* inoculation gave the greatest mean values of pod weight (94 and 91 g/plant^−1^), seed weight (75 and 73 g/plant^−1^), and shelling (79.78% and 80.22%) of peanut crop in both growing seasons, respectively, whereas no significant difference was obtained using *Bradyrhizobium *sp. and *Azotobacter *sp. with or without plant residues, in both seasons. These results are in line with those obtained by Badawi et al., 2011. There was a clearly significant effect with or without plant residue application, as shown in Table 2. These results are in close agreement with Abd El-Moez (1995), and Zaki et al. (2017), who found that the application of plant residues in sand soil increased plant height and dry matter of wheat, as well as nitrogen content in soil.

**Table 2:** Fresh weight and dry weight after 75 DFS, pod weight, seed weight, shelling, and 100-seed weight as affected by the interaction between bio-fertilizer and plant residue treatments in the two growing seasons

	Plan tresidues	Bio-fertilizer	No. of nodule	Fresh weight (g/m^2^)	Dry weight (g/m^2^)	Pod weight g/plant	Seed weight g/plant
1st season	With plant residues	*Bradyrhizobium *sp.	166.0	2080	349	94	75
		*Azotobacter sp.*	23.3	1951	331	92	72
		Uninoculated	10.3	1665	312	83	59
	Without plant residues	*Bradyrhizobium *sp.	112.3	1805	301	86	66
		*Azotobacter *sp*.*	21.7	1681	289	82	60
		Uninoculated	10.7	1071	179	47	29
		L.S.D. at 0.05	3.82	93.30	48.42	4.17	7.94
2nd season	With plant residues	*Bradyrhizobium *sp	147.0	2060	335	91	73
		*Azotobacter *sp*.*	31.0	1915	325	89	70
		Uninoculated	20.3	1528	273	81	56
	Without plant residues	*Bradyrhizobium *sp	98.3	1767	297	90	67
		*Azotobacter *sp*.*	31.0	1626	280	85	61
		Uninoculated	20.3	1003	172	49	31
		L.S.D. at 0.05	3.43	88.82	29.40	5.12	10.29

100- seed weight of peanut with different fertilization treatments and inoculation is presented in Table 2. It was obvious that the application of both with or without plant residues resulted in higher 100- seed weight when inoculated with *Bradyrhizobium *sp*.* and *Azotobacter *sp*.* inoculation compared to those of other through the two seasons. In this respect, there is no significant difference between bacterial inoculated treatments. Plant residues caused increments in 100- seed weight by about 12.2% and 11%, as well as 10.7% and 6.0% over or beneath the uninoculated control for *Bradyrhizobium *sp*.* and Azotobacter sp*.* inoculation, in both growing seasons, respectively. Another trend was noticed with treatments without plant residues, which caused increments in 100- seed weight by about 45.6% and 43.1%, as well as 35.9% and 29.7% over or under the uninoculated (control) for *Bradyrhizobium *sp*.* and *Azotobacter sp.* inoculation in the 1st and 2nd seasons, respectively. The outcome confirmed inoculation with *Bradyrhizobium *sp. with plant residue application achieved the greatest values of 100-seed weight 92 g/ plant^−1^ in the first season, and 93 g/ plant^−1^ in the 2nd, respectively. In this respect, there was no clear significant difference between *Bradyrhizobium *sp. as symbiotic nitrogen fixing bacteria, and *Azotobacter *sp. as asymbiotic nitrogen fixing bacteria. However, there is a significant difference between inoculated and uninoculated treatments with or without plant residues during the two seasons. This result might be attributed to the rise of plant residues in soil, which improved the soil structure and nutrient supply to plants. Similar effects were obtained by Mohamed (1994) and Metwally et al. (1998).

In both growth seasons, bacterial inoculation with or without plant residues had a substantial effect on (seed yield kg ha^−1^), (straw yield kg ha^−1^), and biological (yield kg ha^−1^) (Table 3). In the first and second seasons, applying *Bradyrhizobium *sp. with plant residues significantly increased seed yield (42.25% and 34.15%, respectively), straw yield (10.04% and 14.01%), and biological yield (22.11% and 14.01%, respectively) compared to the uninoculated treatment. Moreover, with *Bradyrhizobium *sp. inoculation and without plant residue application, the increments reached 95.04% and 88.38% in seed yield, 30.06% and 39.43% in straw yield, and 51.83% and 56.50% in biological yield, in the two growing seasons respectively, compared to the uninoculated plants. The increase in grain and straw yield might be attributed to the improved nitrogen availability in the soil, which resulted in greater growth, development, and yield (Erman et al., 2011).

**Table 3:** Seed yield, straw yield, and biological yield as affected by the interaction between bio-fertilizer and plant residue treatments in the two growing seasons

	Plant residues	Bio-fertilizer	Seed yield (kg ha^−1^)	Straw yield (kg ha^−1^)	Biological yield (kg ha^−1^)
1st season	With plant residues	*Bradyrhizobium *sp.	4648	6312	10,987
		*Azotobacter *sp*.*	4478	6028	10,507
		Uninoculated	3261	5736	8997
	Without plant residues	*Bradyrhizobium *sp.	4291	5853	10,173
		*Azotobacter *sp*.*	4020	5275	9309
		Uninoculated	2200	4500	6700
		L.S.D. at 0.05	241.7	181.2	294.0
2nd season	With plant residues	*Bradyrhizobium *sp.	4529	6211	10,740
		*Azotobacter *sp.	4380	5992	10,372
		Uninoculated	3376	5448	8824
	Without plant residues	*Bradyrhizobium *sp.	4348	6000	10,348
		*Azotobacter *sp.	4113	5325	9439
		Uninoculated	2308	4303	6612
		L.S.D. at 0.05	160.7	140.1	230.8

As demonstrated in Table 4, the oil content in peanut was significant in the two growing seasons. The oil content of peanut treatments was between 46.20 and 52.29% based on dry weight. The best oil content 52.29% in the 1st season, and 52.21% in the 2nd season was reported in *Bradyrhizobium *sp. inoculation with plant residue application, whereas the lowest (46.20%) was in uninoculated without plant residue application. The carbohydrate percentage of seed peanut reached 18.39% in the first season, and 18.65% in the 2nd season, as a result of uninoculated without plant residues. Peanut seeds contain 9.5–19.0% carbohydrate on a dried seed basis. It is an excellent supply of mineral (P, Ca, Mg and K), and vitamins (E, K and B group). Peanuts is also an inexpensive source of protein, a great source of essential vitamins and minerals, and a part of many food products (Chamberlin et al., 2014; Chowdhury et al., 2015). Bacterial inoculation plays an essential role in the assimilation of plants, which appears in its improvement of such characteristic. It may also be related to the role of plant phytohormones, such as IAA, GA, and CKS, which promote plant growth, and cell division, breaking the special dominances, hence, encouraging photosynthesis, and assimilating accumulation (Zaki et al., 2012).

**Table 4:** Oil %, total carbohydrate content %, seed protein %, and straw protein percentage as affected by the interaction between bio-fertilizer and plant residue treatments in the two growing seasons

	Plant residues	Bio-fertilizer	Oil (%)	Total carbohydrate (%)	Seed Protein (%)	Straw protein (%)
1st season	With plant residues	*Bradyrhizobium *sp.	52.29	14.95	28.68	16.34
		*Azotobacter *sp.	50.44	15.93	28.37	16.09
		Uninoculated	44.78	16.69	25.37	13.69
	Without plant residues	*Bradyrhizobium *sp.	51.40	17.27	26.26	14.97
		*Azotobacter *sp*.*	48.21	17.70	25.86	15.26
		Uninoculated	46.20	18.39	20.63	10.85
		L.S.D. at 0.05	0.14	0.87	0.39	0.77
			52.21	15.13	28.57	15.89
2nd season	With plant residues	*Bradyrhizobium *sp.	50.89	16.28	27.98	15.67
		*Azotobacter *sp.	45.23	16.94	25.22	13.27
		Uninoculated	51.11	16.85	26.83	15.20
	Without plant residues	*Bradyrhizobium *sp.	49.24	16.94	25.40	14.96
		*Azotobacter *sp.	46.51	18.65	21.21	11.07
		Uninoculated	0.45	0.74	0.64	0.85
		L.S.D. at 0.05	52.21	15.13	28.57	15.89

In the first and second seasons, *Bradyrhizobium *sp. inoculation with plant residues produced the greatest seed protein percentages at 28.68%, and 28.57%, respectively, and the best straw protein percentages at 16.34% and 15.89%. In the two seasons, uninoculated and without plant residue application yielded the lowest protein percentage in peanut seeds (20.63% and 21.21%), and straw (10.85% and 11.07%). This means that utilizing *Bradyrhizobium *sp. as a biofertilizer increased not only the peanut yield and quality, but also its nutritional content. This outcome could be due to nitrogen's favorable aftereffects on metabolism and growth, which are frequently observed. The application of Rhizobia with Enterobacter resulted in the greatest increase in production, and its component in relation to organic matter (chicken manure), which might be attributed to the organic fertilizer's two effects. Organic manure provides nutrient-dense organic carbon for microbial biomass, which converts unavailable nutrients in organic matter to available nutrients, thus boosting soil microbial populations. These findings are consistent with those of (Siam et al., 2013, 2015; El-Quesni et al., 2010). The initial impact of organic manure fertilizer might be triggered as a final product of improving the physical, chemical, and biological characteristics of the sandy soil. These results are in agreement with those obtained by Rizk et al. (2012).

Total nitrogen uptake varied with inoculation and plant residues treated through the two seasons. *Bradyrhizobium *sp. and *Azotobacter *sp. inoculated without plant residues showed higher total N uptake than those managed with uninoculated in the 1st season, without any difference during the 2ng season (Table 5). Throughout both seasons, the highest values were recorded with *Bradyrhizobium *sp. and plant residues in the 1st season. This clearly suggests that the inoculation of *Bradyrhizobium *sp. or *Azotobacter *sp. enhanced the nitrogen fixation, and made an optimistic contribution in facilitating a much better uptake of nitrogen from the soil, and the applied fertilizer. The increase in nitrogen from nitrogen fixation, and soil nitrogen uptake in inoculated plant agree with the results by Butler and Ladd (1985).

**Table 5:** N-uptake and Nitrogen status in peanut plant after 75 DFS, as affected by the interaction between bio-fertilizer and plant residue treatments in the two growing seasons

	Plant residues	Bio-fertilizer	*N*-uptake (kg ha^−1^)	Ndff (%)	Ndfs (%)	N dfa (%)	N- fixed (kg ha^−1^)
1st season	With plant residues	*Bradyrhizobium *sp.	169.7	16.72	20.14	63.14	17.90
		*Azotobacter *sp.	155.7	16.98	20.46	62.56	16.51
		Uninoculated	123.3	39.92	48.09	11.99	5.90
	Without plant residues	*Bradyrhizobium *sp.	139.4	21.92	25.79	52.28	15.9
		*Azotobacter *sp.	126.1	21.97	25.86	52.18	14.43
		Uninoculated	55.73	40.58	47.92	11.66	2.64
		L.S.D. at 0.05	21.6	0.24	0.64	0.51	1.46
2nd season	With plant residues	*Bradyrhizobium *sp.	163.7	16.04	17.75	66.20	17.38
		*Azotobacter *sp.	149.6	16.36	18.11	65.52	16.03
		Uninoculated	107.5	41.94	46.43	11.63	5.16
	Without plant residues	*Bradyrhizobium sp*	129.9	21.58	23.72	54.69	15.31
		*Azotobacter *sp.	119.6	23.30	25.44	51.25	14.28
		Uninoculated	51.6	42.42	46.32	11.25	2.45
		L.S.D. at 0.05	11.1	0.42	0.79	0.91	1.15

To investigate whether this nitrogen increase was the consequence of the achieved improvement of the nitrogen fixation by symbiotic and asymbiotic nitrogen fixing bacteria, the sum total nitrogen in inoculated and uninoculated peanut plants was fractionated into nitrogen derived from soil (Ndfs), nitrogen derived from fertilizer (Ndff), nitrogen derived from atmosphere (Ndfa), and fixed nitrogen. Nitrogen derived from air (Ndfa) by plant showed higher values with *Bradyrhizobium *sp*.,* followed by *Azotobacter *sp. in the 1st season (Table 5). Inoculation *Bradyrhizobium *sp. with application of plant residues resulted in a significant increase in nitrogen derived from air, except in uninoculated plants. These results may be caused by the symbiotic relationship of *Bradyrhizobium *sp. or *Azotobacter *sp., since the asymbiotic relationship with the roots of peanut crop fixed the atmospheric nitrogen into the peanut roots; thus, the yield increased. This clearly suggests that the inoculation of peanut with *Bradyrhizobium *sp. or *Azotobacter *sp. did not only enhance the nitrogen fixation, but also had a positive contribution in facilitating a better uptake of nitrogen from the soil, and the applied fertilizer. A sharp decrease in Ndfa, and a greater dependency on nitrogen from the soil were observed in both seasons with the uninoculated treatment, indicating that the presence of native rhizobium in soil was not adequate for biological nitrogen fixation. In another experiment, inoculation of *C. caeruleum* and *Pueraria* with various strains of *Bradyrhizobium* also could not increase its growth. In some cases, crop failures occur due to the lack of appropriate inoculant (Wahab et al*.* 1989). Rizkalla et al*.* (2014) indicated that percentages and absolute values of nitrogen derived from organic residue inoculated chickpea were really low compared to nitrogen derived from air (Ndfa), and N derived from mineral fertilizer (Ndff).

Portions and nitrogen uptake values of nitrogen derived from fertilizer (Ndff) % in peanut plant are presented in Table 5. Results clarified that % Ndff under different treatments of plant residue additions increased with uninoculated, in comparison with *Bradyrhizobium *sp. and *Azotobacter *sp. through the two seasons. There was no significant difference between *Bradyrhizobium *sp. and *Azotobacter *sp. (21.92% and 21.97%, respectively). However, the Ndff % in this study was similar to that obtained in cowpea grown in field conditions (Sarr et al., 2008).

The best values of nitrogen derived from soil (Ndfs) (48.09% and 47.92%) were obtained by uninoculated treatment, received with or without plant residue addition in the 1st season. The percentage of Ndfs in uninoculated was significantly higher than in the inoculated treatment. This is especially important since the uninoculated treatment required more nitrogen from other sources, such as applied nitrogen fertilizer, or plant residues, compared to inoculated treatments (*Bradyrhizobium *sp. or Azotobacter sp.) that fixed atmospheric nitrogen as another source of nitrogen. High inorganic N concentrations were proven to inhibit nitrogen fixation (Streeter & Wong, 1988).

The contribution of the N- fixed in the plant of inoculated peanut was significantly higher than in uninoculated peanut. The nitrogen derived from N- fixed recorded 5.16 kg ha^−1^ in uninoculated peanut, 17.90 kg ha^−1^ in *Bradyrhizobium *sp. inoculated, and 16.15 kg ha^−1^ *Azotobacter *sp. treatment with plant residue application. Such superiority in yield and yield components from treating peanut seeds with *Bradyrhizobium *sp. inoculation might be caused by nitrogen fixation, which had a noticeable influence on the growth of peanut plants, and improved yield and yield components. Nitrogen is an essential factor in achieving better growth and development of the vegetative and reproductive organs of peanut, increasing photosynthesis rate and photosynthetic matter production, and consequently the yield components and seed yield of peanut. The values corresponded to the quantity of nitrogen per source (soil, fertilizer, and atmosphere). No significant difference was observed between *Bradyrhizobium *sp. inoculated, and *Azotobacter *sp. inoculated in the 1st season. Regarding nitrogen derived from fertilizer (Ndff), nitrogen derived from soil (Ndfs), and nitrogen derived from fertilizer (Ndff), no significant difference was noticed between *Bradyrhizobium *sp. and *Azotobacter *sp. inoculated, without plant residues in the second season. Some studies demonstrated that plants had a tendency to extract lesser nutrients from the soil stock when other sources are available. In this regard, Rees et al*.* (1993) reported a decreased uptake of soil nitrogen following the application of legume residues with a higher nitrogen content. Robinson (2001) concluded that the natural abundances of the rare stable isotope of nitrogen, ^15^N, is now widely used in research on nitrogen cycling, in organisms and ecosystems.

## Conclusion

Effective inoculation with *Bradyrhizobium *sp*.* strain stimulated the growth of field grown peanut, and increased the total N amount, by increasing the amounts of nitrogen obtained from symbiotic nitrogen fixation, and the soil. The low contribution of plant residues in nitrogen nutrition in peanut could be attributed to the slow release and decomposition of plant residues. This study recommends placing plant residues in the soil long before planting, to be allowed the time needed for decomposition and conversion of organic nitrogen. For subsequent cultivation, the present research topic has particular importance for the management of soil fertility in Egypt, which is one of the limiting factors in agricultural production.

## Data Availability

The datasets used in this study are available from the corresponding author on reasonable request
